# COVID-19 vaccination implementation in six lower- and middle-income countries: Successes, challenges, and lessons for pandemic preparedness

**DOI:** 10.1371/journal.pgph.0004417

**Published:** 2025-05-07

**Authors:** Cara Tupps, Dora Curry, Amanda Edwards, Eva Bazant, Ann Moen, Anthony W. Mounts, Joseph Bresee

**Affiliations:** The Task Force for Global Health, Decatur, Georgia, Unites States of America; Fundacao Oswaldo Cruz, BRAZIL

## Abstract

The COVID-19 pandemic challenged health systems in low- and middle-income countries (LMIC) to rapidly deploy vaccines, target adult populations, and integrate the COVID-19 vaccine into existing vaccination programs. This evaluation examined COVID-19 vaccination implementation and planning experiences of six LMICs. We aimed to identify common strategies and investments contributing to country-level readiness to scale up COVID-19 vaccination and gaps in pandemic preparedness. In-depth interviews were conducted with national COVID-19 vaccination program representatives from Côte d’Ivoire, Kyrgyzstan, Moldova, Pakistan, the Philippines, and Zambia. Interview questions covered activities, barriers, and facilitators related to vaccine integration; planning and financing; digital systems; vaccine infrastructure and delivery; adult immunization; the health workforce; and demand and communications. We used the framework analysis method to establish key themes from the recorded data and categorize our results. Countries with influenza vaccine platforms leveraged these to reach adults with COVID-19 vaccines. Community-based platforms were viewed to be an effective approach to vaccinate prioritized populations. Stand-alone delivery platforms and data systems for COVID-19 vaccination were viewed as inefficient and not cost-effective, and vaccine supply delays and shortages were a major issue. Participants highlighted that integrated planning, management, and financing for vaccination activities facilitated the COVID-19 vaccine roll out, and that National Immunization Technical Advisory Groups filled a gap by providing guidance on prioritizing populations for vaccination. Health workers were viewed as key influencers of vaccine uptake by patients and their vaccination was believed by participants to improve public trust in COVID-19 vaccines. These findings informed the following priority areas for targeted investment and technical support. 1. Improve vaccine procurement and supply. 2. Integrate financing and management of national vaccination programs broadly. 3. Digitize and integrate data systems. 4. Build health workforce capacity. 5. Establish and expand adult and life-course vaccination, including health workers. 6. Address hesitancy and misinformation.

## Introduction

The COVID-19 pandemic challenged resource-limited health systems in low- and middle-income countries (LMIC), with considerable consequences for population health [1–6]. A key challenge was ensuring that COVID-19 vaccines reached adults and vulnerable populations in countries without pre-established platforms for vaccinating these groups. Ministries of Health faced urgent decisions regarding financing COVID-19 vaccination, establishing policies and guidelines, and coordinating delivery [7–9]. Efforts to integrate COVID-19 vaccination with other health programs (e.g., Expanded Program on Immunization (EPI), seasonal influenza vaccination) also presented opportunities and challenges for LMICs. In this context, we refer to integration as the adoption of COVID-19 vaccination into national immunization programs and/or other health services and coordinating program planning, management, financing, infrastructure, implementation, monitoring, and reporting

This evaluation examined the experiences of six LMICs (Côte d’Ivoire, Moldova, Kyrgyzstan, Pakistan, the Philippines, and Zambia) with COVID-19 vaccine planning and implementation before, during, and after the pandemic. The aims were to: 1. Determine specific investments made before and during the pandemic which contributed to preparedness. 2. Identify successes and challenges from countries integrating COVID-19 vaccination into other health programs and future plans for integration [10]. 3. Develop recommendations for policy prioritization and investments that contribute to country-level readiness to scale up COVID-19 vaccination and respond to vaccine-preventable epidemics and pandemics.

This evaluation provides key informant qualitative insights into experiences on LMIC implementation of COVID-19 vaccination. Existing literature focuses on the initial phases of vaccine roll-out and relates to specific areas, such as vaccine delivery [8,11,12], health service utilization, [1–6] vaccine demand and supply, [9,12] and vaccine inequity [7,13,14]. We sought to deepen understanding of country experiences across these and additional thematic areas relevant to vaccine planning and implementation [8,15–17]. Our findings present new insights into integrating COVID-19 vaccination with influenza and adult vaccination.

## Methods

### Country selection

We began with a list of countries which were already engaged with the Task Force for Global Health (TFGH) for COVID-19 and influenza vaccination program support. Six countries -- Côte d’Ivoire, Kyrgyzstan, Moldova, Pakistan, the Philippines, and Zambia -- were ultimately included with the intent to gather a range of perspectives across regions, income classifications, and the presence or absence of influenza vaccination programs. See Table 1 for a summary of country population, economic, and influenza vaccination program characteristics. These countries are in the WHO African, Eastern Mediterranean, European, and Western Pacific regions, and meet lower-middle or upper-middle income classifications according to the World Bank [18]. Inclusion was also dependent upon the country vaccine team’s willingness to participate in key informant interviews and opportunities to conduct interviews in person within the period of data collection.

**Table 1:** Summary of country population and economic characteristics and influenza vaccination program details.

Country	Population – 2022 [23] in millions (M)	Gross national income per capita, Atlas method - 2022, current US$ (income classification) [24]	National influenza vaccine policy, 2019–2022 [20]	Influenza vaccine recommended for at least three target groups, including healthcare workers, 2019–2022 [20]	Partnership for Influenza Vaccine Introduction partner country [21]
Côte d’Ivoire	28.16 M	$2,620 (lower-middle)	Yes (public sector)	Yes	Yes
Kyrgyz Republic	6.97 M	$1,440 (lower-middle)	Yes (public and private sectors)	Yes	Yes
Pakistan	253.82 M	$1,560 (lower-middle)	No data	No data	No
Philippines	115.56 M	$3,950 (lower-middle)	Yes (public sector)	Older adults only	No
Republic of Moldova	2.59 M	$5,500 (upper-middle)	Yes (public sector)	Yes	Yes
Zambia	20.02 M	$1,240 (lower-middle)	No	No data	No

We categorized countries as having a comprehensive influenza vaccine program if they had both a national influenza vaccine policy and official recommendations for influenza vaccination for three or more priority groups as identified by the WHO Strategic Advisory Group of Experts on Immunization (SAGE) [19]. Three eligible countries (Côte d’Ivoire, Kyrgyzstan, and Moldova) have comprehensive influenza vaccination programs per influenza vaccination policy data available through the WHO immunization portal [20]; these countries also take part in the Partnership for International Vaccine Initiatives (PIVI), a Task Force for Global Health initiative which supports the development and sustainability of national influenza vaccination programs [21,22]. (See Table 1.) The Philippines targets older adults for influenza vaccination but did not meet these criteria for having a comprehensive program. Pakistan and Zambia do not have adult influenza vaccination programs.

### Data collection

Primary qualitative data were collected between October 10th and December 7th, 2023. A total of nine COVID-19 vaccination program leaders and EPI program managers participated in six key informant interviews (five in-person and one virtual) The interviewers followed a semi-structured questionnaire and took detailed notes and audio recordings. Participants were asked to describe:

1. Country investments in COVID-19 vaccine planning and implementation made before and during the pandemic
2. Experiences with integration, the healthcare workforce, policy, budgeting, planning and management, delivery mechanisms, communications, digital health systems, and the cold chain
3. Planned investments, policies, and activities related to ongoing COVID-19 vaccination

Interviews were led by in-country and regional consultants employed by TFGH supporting the Ministries of Health with vaccination implementation and evaluation. Interviews were conducted in local languages or in English with interpretation as needed. Transcripts of non-English language interviews were translated into English using professional translation services.

### Ethics approval and consent to participate

This evaluation was reviewed and classified as non-human subjects research by the Centers for Disease Control and Prevention in Atlanta, GA, USA (NCIRD-IE-8/15/22-ab3a6). Representatives from Ministries of Health in each selected country gave verbal or written approval for their country’s representation in the evaluation, and recommended interview participants based on their experiences with COVID-19 vaccination. Questionnaires were translated and shared with participants ahead of interviews, and all participants gave verbal consent to be interviewed and recorded for the purpose of data analysis.

### Data analysis

We used the framework analysis method to establish key themes and categorize our results [25]. An initial conceptual framework was developed based on the research questions and refined according to themes that emerged during data review. Qualitative thematic analysis was performed using Dedoose version 9.3.7 [26]. Data were independently coded by two researchers and reviewed and reconciled into a single set of codes by both researchers. A matrix of common themes, sub-themes, and key findings was developed to record frequency of code usage in interviews.

## Results

The main themes included supply chain, policy and planning, financing and management, data management and use, workforce, vaccine delivery strategies, demand and communications, and safety.

**Fig 1:**
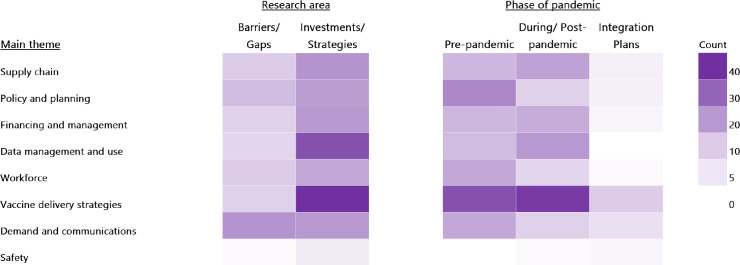
Frequency of code usage by major theme, research areas, and phase of pandemic.

Fig 1 provides a visual representation of the frequency of code usage across these main themes, research questions, and phase of the pandemic.

The strategies and investments which facilitated COVID-19 vaccination, as well as the challenges and gaps described by interview participants, can be broadly grouped into the following programmatic areas:

1. Addressing procurement and global supply issues, including strengthening the cold chain
2. Utilizing NITAGs to inform national policies for new and novel vaccines
3. Integration of vaccine delivery, planning, management, and financing
4. Digitizing data collection and developing interoperable data systems
5. Leveraging health worker vaccination programs to influence public acceptance of the vaccine
6. Use of community-based platforms for vaccine delivery
7. Conducting information, education, and communications campaigns

Country program experiences across these seven areas are described below. (Vaccine safety was infrequently mentioned by participants, and therefore this theme is not covered in detail in this article.)

### 1. Addressing procurement and global supply issues, including strengthening the cold chain

Participants from five countries described critical investments in cold chain improvements and vaccine storage systems during the pandemic. The Zambian participant also described cold chain capacity as a potential barrier to their plans to administer COVID-19 vaccines at HIV and tuberculosis clinics. They discussed the tradeoffs of adding cold chain equipment to these facilities versus training teams of mobile vaccinators to visit facilities without cold chain capacity.

All participants reported vaccine shortages and procurement challenges, especially early in the roll-out of COVID-19 vaccines. The Pakistan participant stated: “The vaccines were supposed to be delivered by January 2021 … we received only 100,000. In the Pakistani context, (that) is just like peanuts. … We were struggling a lot in terms of acquiring the vaccines. … The target was huge, millions, and the supply was not that much.” The Philippines participant also noted that the absence of clear global guidance for prioritizing populations compounded difficulties in utilizing limited vaccine supplies.

Participants from four countries noted that future planning for COVID-19 vaccination is challenged by a lack of vaccines; at the time of the interviews, they were awaiting further guidance from COVAX and Gavi before developing procurement and financing plans.

### 2. Utilizing NITAGs to inform national policies for new and novel vaccines

Participants in Kyrgyzstan, Moldova, and Zambia stated that National Immunization Technical Advisory Groups (NITAGs) played a key role in creating guidelines for prioritizing populations amidst low vaccine supply and filled a gap when global guidance on prioritization was lacking. A Kyrgyzstan participant noted, “Everything was new with the COVID vaccine. Acceptance of a new vaccine was a challenge. Communicating evolving information across different brands, all with different approaches for supply chain and management and logistics, was a major challenge. So you need to have strong systems across all areas in place in order to be prepared for that.” In the absence of a NITAG, participants in the Philippines described challenges due to conflicting national guidelines released by the COVID-19 and routine immunization divisions.

### 3. Integration of vaccine delivery, planning, management, and financing

Participants described integrating COVID-19 vaccination through co-delivery or leveraging pre-pandemic platforms for other vaccinations and health services (Co-delivery is also discussed in area 7 below). We define ‘co-delivery’ in this context as the administration of more than one antigen at the same time or using the same platform. The Philippines participant described using service delivery sites for HIV, tuberculosis, EPI, and maternal health programs for COVID-19 vaccination. Participants also described inefficiencies where they did not or were not able to leverage existing health service delivery platforms. Participants from Côte d’Ivoire stated that an initial strategy of establishing dedicated centers for COVID-19 vaccination was expensive and inefficient. “We wanted to put in place new structures to achieve (targets), but very quickly, we understood that this was a bad strategy, and we involved the districts and regions, including all the health centers.”

Participants noted that the presence of adult immunization programs impacted the effectiveness of countries’ implementation of COVID-19 vaccination. Kyrgyzstan and Moldova participants stated that adult influenza vaccination platforms were utilized for COVID-19, and planning, management, and financing processes were and will continue to be integrated. Participants from these two countries believed that integration of the COVID-19 and influenza programs contributed to a more streamlined COVID-19 vaccine roll-out. In Kyrgyzstan, the EPI and National Influenza Immunization Programs coordinated to submit a single budget request, develop a joint allocation plan, and conduct joint microplanning for influenza and COVID-19 vaccination. Moldova aligned processes for purchasing COVID-19 vaccination with its existing procedure for routine vaccination, including influenza, and noted that future procurement for COVID-19 vaccine will use the same funding source as influenza.

When asked about their plans for integrating COVID-19 into their current systems, participants in three countries mentioned having separate management structures for different vaccines as a barrier. In the Philippines, the participant noted there was debate about which program should manage COVID-19 vaccination since childhood vaccination, influenza, human papillomavirus (HPV), and other vaccines are all managed by separate departments. The participant in the Philippines stated that the country plans to transition to life-course vaccination, but that a strategy is lacking for how to move from an antigen-specific approach to integrated vaccine management. In Pakistan, the participant mentioned that they would like to merge COVID-19 and EPI with other health programs, such as maternal health and nutrition, but that there were challenges due to different target populations, supply chain processes, and capacity-building needs. A participant from Côte d’Ivoire stated that some health programs still operate independently from EPI, which makes integration difficult.

Participants noted that plans for financing COVID-19 vaccination are limited by requirements to use funds for specific vaccine initiatives rather than across national vaccine systems. The Philippines participant stated: “Identifying resource opportunities and financing is initiated by each division, and (we) don’t know whether funding (we) apply for and receive will only be for a single antigen or whether it can be used for all immunization activities.” The participant in Pakistan stated that funding for the national management structure that oversees integrated vaccination campaigns was dwindling. A quote from Kyrgyzstan exemplifies country views that holistic financing is needed: “

There really cannot be prioritization of investment for preparedness for COVID-19 or something else in the future. (We) need investment across the entire system; all areas are equally important.”

### 4. Digitizing data collection and developing interoperable data systems

Data systems were mentioned as a key investment and/or barrier by participants from all countries. Participants from four countries described the development of digital systems for COVID-19 vaccination which facilitated monitoring and tracking efforts. Kyrgyzstan’s participants described how the country transitioned COVID-19 vaccination system into a single electronic data system encompassing childhood and maternal vaccines including EPI, HPV, and hepatitis B, which merged health record data, diagnostics, and adverse events following immunization. Participants from Côte d’Ivoire, Moldova, and the Philippines noted efforts to combine existing vaccine or health record information systems. Zambian participants described plans for merging data systems: “The COVID tracker data is interoperable with DHIS2 data and already integrated. It helps to track defaulters, follow up for subsequent doses, and generate certificates.... So far, the COVID-19 tracker is limited to COVID-19 data only. The plan is underway to expand the system so we can also begin collecting individual data for other vaccines.”

Participants from four countries noted challenges of managing separate data systems to track COVID-19 vaccination, influenza, HPV, and EPI vaccines. Several participants stated that their country’s vaccine databases are not interoperable with those for routine health records or disease surveillance. Participants described investments to map data systems and improve interoperability; however, this process is time-consuming and limited by funding, technical capacity, and program management structures.

### 5. Leveraging health worker vaccination programs to influence public acceptance of the vaccine

Participants from all countries stated that they prioritized healthcare worker vaccination for COVID-19; they felt that this increased public trust and made healthcare workers more effective in encouraging uptake of the vaccine. A participant from Kyrgyzstan noted, “There is a lot of trust in and respect for medical providers, and the public will by and large do what is recommended to them by doctors and nurses…one thing that was successful was that health workers were vaccinated first.”

Kyrgyzstan, Côte d’Ivoire, and Moldova have national health worker vaccination programs for seasonal influenza. Participants from Kyrgyzstan and Moldova reported their seasonal influenza vaccination programs for health workers facilitated efforts to vaccinate this group against COVID-19. Côte d’Ivoire participants stated that health workers are vaccinated against hepatitis B and meningitis in addition to influenza, but that the scale of health worker vaccination activities was limited prior to the pandemic. In Moldova, participants mentioned that health worker sensitization efforts for influenza vaccination prior to the pandemic increased their acceptance of the COVID-19 vaccine.

Participants from five countries described investment in health workforce training and capacity during the pandemic. Activities included sensitizing healthcare workers to the best practices and guidelines for COVID-19 vaccine administration, storage, and safety of new vaccines. Zambia plans to leverage its success and lessons learned in training health workers during the pandemic for other vaccines.

All participants described systemic issues within the healthcare workforce including high turnover, burnout, and low recruitment rates. The Côte d’Ivoire participant noted that redirecting limited workforce capacity from routine care to COVID-19 response created gaps in staffing that further strained country health systems. Participants described various approaches to addressing these limitations, including investing in a surge capacity roster in Zambia and Côte d’Ivoire and bonuses to combat turnover in Kyrgyzstan.

### 6. Use of community-based platforms for vaccine delivery

Participants from three countries described successfully utilizing community-based COVID-19 vaccination and other antigen-delivery platforms. Within this evaluation, community-based platforms were defined as vaccination sites or activities set up outside of traditional healthcare settings such as clinics and hospitals in order to increase accessibility and convenience (e.g., shopping centers), leverage trusted locations (e.g., places of worship), vaccinate large groups of individuals (e.g., mass vaccination events), or target hard-to-reach communities or marginalized populations (e.g., mobile or house-to-house outreach). Participants from Côte d’Ivoire, Moldova, and Pakistan stated their countries utilized a mobile vaccination strategy to deliver vaccines to remote and hard-to-reach populations. In the Philippines, vaccination sites were set up in malls and mosques; the participant stated that the vaccine program plans to use these sites for COVID-19 vaccination and other vaccine campaigns in the future. The vaccine program in Zambia also indicated that it plans to continue to operate community-based vaccine sites for COVID-19, as well as routine immunization. No participants in this evaluation described any challenges related to community-based platforms for vaccine delivery.

### 7. Conducting information, education, and communications campaigns

Participants from all countries described taking steps to address misinformation and hesitancy related to the COVID-19 vaccine, promote transparency, and improve vaccination coverage. Tactics included the use of community influencers, mass and social media campaigns, and information hotlines to improve understanding and acceptance of COVID-19 vaccination. Participants from the Philippines said the country leveraged social mobilization platforms for directly observed treatment for tuberculosis and routine immunization programs for COVID-19 vaccination. Participants from Pakistan said the country plans to maintain its COVID-19 helpline through 2026 and incorporate routine immunization information. Participants from the Philippines indicated the country will apply strategies to reduce hesitancy developed during the pandemic to increase demand. Furthermore, Zambia’s participants described applying lessons learned in addressing COVID-19 vaccine misinformation to its HPV immunization program.

Participants from five countries described skepticism, hesitancy, lack of confidence, and infodemics as barriers to vaccination. The Philippines and Kyrgyzstan participants noted that COVID-19 vaccine preferences (e.g., mistrust of specific brands and belief that certain vaccines were inferior in quality) limited the ability to effectively utilize limited vaccine supplies.

Participants from two countries expressed caution about co-delivery of different vaccines due to side effects and hesitancy. A participant from Kyrgyzstan noted concern that co-administration of antigens may harm vaccine coverage if people associate COVID-19 post-vaccination symptoms with the influenza vaccine. Therefore, the country program spaces COVID-19 and influenza vaccinations by 7–14 days. A participant in Zambia also stated that hesitancy and misinformation related to the COVID-19 vaccine may negatively affect coverage for other vaccines if they are co-delivered.

## Discussion

Our findings were used to develop the following recommendations for countries planning and implementing COVID-19 vaccination, including integration with national immunization programs. These recommendations are also aimed at funders and implementing partners focused on bolstering country health systems and pandemic preparedness; investment in these areas can improve the targeting of donor funding and technical support to LMIC towards areas of need as described by the countries themselves. These recommendations are ordered from macro-/global-level recommendations, followed by national level, then individual level.

1. Improve vaccine procurement and supply
2. Integrate financing and management of national vaccination programs broadly
3. Digitize and integrate data systems
4. Build health workforce capacity
5. Establish and expand adult and life-course vaccination, including health workers
6. Address hesitancy and misinformation
7. Improve vaccine procurement and supply. Lack of access to new vaccines in LMIC is a significant barrier to pandemic preparedness that must be addressed through global advocacy for vaccine equity and policy change. [27] Establishing new partnerships and mechanisms to facilitate vaccine access in LMIC is a crucial area for advocates and policymakers. In this post-pandemic phase, we must ensure that countries can continue accessing COVID-19 vaccines and encourage transparency of costs and capacity by the pharmaceutical industry to ensure vaccines are priced appropriately and affordably. Furthermore, this is an opportunity to develop better and more equitable procurement processes that enable swift access to novel vaccines for all countries, regardless of purchasing power, in future public health emergencies.
8. Integrate financing and management of national vaccination programs broadly. Significant resources will be required in LMIC for integrated planning, policy development, budgeting and procurement, data management, implementation, and monitoring of adult and childhood vaccination programs [8,15–17]. As underscored by our findings, funding and support for integrated vaccination approaches in LMIC should be a key area of focus for global funders and technical support partners, including investment in the broader vaccine infrastructure and guidance for transitioning from vertical to horizontal management structures.
9. Digitize and integrate data systems. Investment in the digitization of data and strengthening of national health information systems to improve vaccination planning and monitoring is key to pandemic preparedness. [28] Investments in vaccine data systems should ensure interoperability with existing systems, adaptability to meet new needs, and availability of multiple data streams for decision-making within a single system. Furthermore, improvements to cold chain and vaccine delivery systems are needed as countries move towards routine delivery of COVID-19 vaccination and co-delivery with other antigens [12]. Our results demonstrate the need to invest in integrated systems to support upgrades and maintenance for both digital and physical infrastructure.
10. Build health workforce capacity. Strengthening the healthcare workforce is a critical component of pandemic preparedness. Burnout and turnover, especially in low-resource settings, are pervasive issues compounded by the effects of the COVID-19 pandemic [29]. Healthcare workers serve a crucial role in influencing populations to accept vaccination and are more likely to recommend the vaccine to patients when vaccination and sensitization efforts targeting healthcare workers are in place [30–37]. A comprehensive approach to supporting the health workforce requires sufficient capacity building, training, sensitization, and compensation.
11. Establish and expand adult and life-course vaccination, including health workers. This evaluation found that the experience of implementing COVID-19 vaccination strengthened mechanisms to reach adult populations, which could make it easier to expand adult vaccination programs in LMICs. The presence of a national seasonal influenza program has been described as contributing to country pandemic preparedness [38]. However, LMIC are less likely to have adult immunization or influenza programs compared to upper-middle and high-income countries [39,40]. Investment and technical support to countries to expand adult immunization programs and life-course vaccination is needed to strengthen this essential pillar of pandemic preparedness [41].

Vaccinating health workers is crucial to protecting the health workforce during epidemics and pandemics. Furthermore, health worker vaccination provides countries with experience in developing systems to provide adult vaccination. The results of this evaluation demonstrate that health worker vaccination should be considered within policy and investment plans to create platforms to vaccinate adults, strengthen health systems, and position health workers to serve as key influencers to increase public acceptance of vaccination.

1. Address hesitancy and misinformation. Ongoing vaccine hesitancy and misinformation surrounding COVID-19 vaccination may present a challenge to delivery and integration [42–44]. As COVID-19 vaccination transitions from emergency to routine use, countries must continue to monitor misinformation, develop national demand-generation plans, and reinforce vaccination as a social norm. As our findings note, this is crucial to the success not only of COVID-19 vaccination efforts but also integrated antigen delivery programs. Integration of communication efforts for other health interventions also presents an opportunity to co-deliver messages about COVID-19 vaccination, such as during seasonal influenza campaigns or routine immunization visits.

## Limitations

The evaluation is limited to the experiences of six countries’ immunization programs and was not representative of all LMICs or any geographic region. We prioritized countries in which the researchers had established relationships with key stakeholders and where consultants were available to conduct interviews in order to maximize the use of limited resources. Though the six countries span four WHO regions, several income levels within middle-income countries, and a variety of sociodemographic contexts which stakeholders in other countries may find relevant to their own settings, the viewpoints were limited to those communicated by one or two national-level stakeholders per country. The broad nature of this survey did not guarantee that all participants could provide feedback for each specific area, and so our results were also limited by gaps in participant knowledge and experiences. We worked to mitigate this by seeking out knowledgeable interviewees that would provide as much detailed country program knowledge as possible, but the results are nevertheless limited in their generalizability due to the study design. Additional limitations to the study’s data arise from the translation of interviews and the sensitive nature of discussing internal challenges with external interviewers. Though we tried to minimize the potential impact of these concerns, there may still have been some impact on the quality, completeness, or accuracy of data received from participants.

Additionally, this evaluation did not use vaccine coverage as a means of verification for what strategies or investments countries described as effective. LMICs were negatively impacted by delayed donations and vaccine shortages that hindered their ability to achieve target coverage rates and were unrelated to choices or policies made by national authorities. For this reason, vaccine coverage may not provide a full or accurate measure of the effectiveness of country-level implementation approaches to COVID-19 vaccination. Instead, the implementation experiences of LMIC in this evaluation complement analyses of factors influencing COVID-19 coverage rates by providing qualitative data and context as described by country program representatives on investments and approaches they felt were most valuable.

## Conclusion

These case studies describe the experiences of six LMIC in implementing COVID-19 vaccination and planning for the transition to routine and integrated delivery. The most important strategies described by participants were addressing procurement issues and strengthening the cold chain, using NITAGs to inform national policy, integrating vaccination, data digitization, leveraging the health workforce to improve vaccine acceptance, using community-based delivery platforms, and conducting effective communication with the public. Ensuring continued and affordable access to COVID-19 vaccines, increasing vaccine equity, and ensuring swift access to novel vaccines for LMIC during global health emergencies is a crucial area for advocates and policymakers to address before the threat of a new pandemic becomes reality. Integration requires breaking down vertical components of immunization program management and planning, integrating digital health information systems, and leveraging improvements to supply and cold chain mechanisms for multiple antigens. Countries with influenza immunization programs that were co-financed and co-managed with other routine immunization activities had strong foundations on which to integrate COVID-19 vaccination. This illustrates the importance of investing in adult and life course immunization platforms to increase resilience for routine vaccine programs and bolster preparedness for future pandemic vaccination initiatives. Strengthening the health workforce must include capacity building for integrated delivery, sensitization, and training in education and communication techniques to increase trust amongst the public and decrease vaccine hesitancy.
