# Correction: COVID-19 vaccination implementation in six lower- and middle-income countries: Successes, challenges, and lessons for pandemic preparedness

**DOI:** 10.1371/journal.pgph.0005447

**Published:** 2025-11-07

**Authors:** The PLOS Global Public Health Staff

In the Discussion section, the list was formatted incorrectly. Please see the correct formatting here,

1. Improve vaccine procurement and supply,
2. Integrate financing and management of national vaccination programs broadly,
3. Digitize and integrate data systems,
4. Build health workforce capacity,
5. Establish and expand adult and life-course vaccination, including health workers
6. Address hesitancy and misinformation

1 Improve vaccine procurement and supply. Lack of access to new vaccines in LMIC is a significant barrier to pandemic preparedness that must be addressed through global advocacy for vaccine equity and policy change. [27] Establishing new partnerships and mechanisms to facilitate vaccine access in LMIC is a crucial area for advocates and policymakers. In this post-pandemic phase, we must ensure that countries can continue accessing COVID-19 vaccines and encourage transparency of costs and capacity by the pharmaceutical industry to ensure vaccines are priced appropriately and affordably. Furthermore, this is an opportunity to develop better and more equitable procurement processes that enable swift access to novel vaccines for all countries, regardless of purchasing power, in future public health emergencies.
2 Integrate financing and management of national vaccination programs broadly. Significant resources will be required in LMIC for integrated planning, policy development, budgeting and procurement, data management, implementation, and monitoring of adult and childhood vaccination programs [8,15–17]. As underscored by our findings, funding and support for integrated vaccination approaches in LMIC should be a key area of focus for global funders and technical support partners, including investment in the broader vaccine infrastructure and guidance for transitioning from vertical to horizontal management structures.
3 Digitize and integrate data systems. Investment in the digitization of data and strengthening of national health information systems to improve vaccination planning and monitoring is key to pandemic preparedness. [28] Investments in vaccine data systems should ensure interoperability with existing systems, adaptability to meet new needs, and availability of multiple data streams for decision-making within a single system. Furthermore, improvements to cold chain and vaccine delivery systems are needed as countries move towards routine delivery of COVID-19 vaccination and co-delivery with other antigens [12]. Our results demonstrate the need to invest in integrated systems to support upgrades and maintenance for both digital and physical infrastructure.
4 Build health workforce capacity. Strengthening the healthcare workforce is a critical component of pandemic preparedness. Burnout and turnover, especially in low-resource settings, are pervasive issues compounded by the effects of the COVID-19 pandemic [29]. Healthcare workers serve a crucial role in influencing populations to accept vaccination and are more likely to recommend the vaccine to patients when vaccination and sensitization efforts targeting healthcare workers are in place [30–37]. A comprehensive approach to supporting the health workforce requires sufficient capacity building, training, sensitization, and compensation.
5 Establish and expand adult and life-course vaccination, including health workers. This evaluation found that the experience of implementing COVID-19 vaccination strengthened mechanisms to reach adult populations, which could make it easier to expand adult vaccination programs in LMICs. The presence of a national seasonal influenza program has been described as contributing to country pandemic preparedness [38]. However, LMIC are less likely to have adult immunization or influenza programs compared to upper-middle and high-income countries [39,40]. Investment and technical support to countries to expand adult immunization programs and life-course vaccination is needed to strengthen this essential pillar of pandemic preparedness [41].

Vaccinating health workers is crucial to protecting the health workforce during epidemics and pandemics. Furthermore, health worker vaccination provides countries with experience in developing systems to provide adult vaccination. The results of this evaluation demonstrate that health worker vaccination should be considered within policy and investment plans to create platforms to vaccinate adults, strengthen health systems, and position health workers to serve as key influencers to increase public acceptance of vaccination.

6 Address hesitancy and misinformation. Ongoing vaccine hesitancy and misinformation surrounding COVID-19 vaccination may present a challenge to delivery and integration [42–44]. As COVID-19 vaccination transitions from emergency to routine use, countries must continue to monitor misinformation, develop national demand-generation plans, and reinforce vaccination as a social norm. As our findings note, this is crucial to the success not only of COVID-19 vaccination efforts but also integrated antigen delivery programs. Integration of communication efforts for other health interventions also presents an opportunity to co-deliver messages about COVID-19 vaccination, such as during seasonal influenza campaigns or routine immunization visits.

The publisher apologizes for the error.
